# Attitudes About Extremely Preterm Birth Among Obstetric and Neonatal Health Care Professionals in England: A Qualitative Study

**DOI:** 10.1001/jamanetworkopen.2022.41802

**Published:** 2022-11-14

**Authors:** Katie Gallagher, Chloe Shaw, Maryam Parisaei, Neil Marlow, Narendra Aladangady

**Affiliations:** 1 EGA Institute for Women’s Health, University College London, London, United Kingdom; 2 Department of Obstetrics and Gynaecology, Homerton Healthcare NHS Foundation Trust, London, United Kingdom; 3 Department of Neonatology, Homerton Healthcare NHS Foundation Trust, London, United Kingdom; 4 Centre for Paediatrics, Barts and the London School of Medicine and Dentistry, QMUL, London, United Kingdom

## Abstract

**Importance:**

Variation in attitudes between health care professionals involved in the counseling of parents facing extremely preterm birth (<24 wk gestational age) may lead to parental confusion and professional misalignment.

**Objective:**

To explore the attitudes of health care professionals involved in the counseling of parents facing preterm birth on the treatment of extremely preterm infants.

**Design, Setting, and Participants:**

This qualitative study used Q methods to explore the attitudes of neonatal nurses, neonatologists, midwives, and obstetricians involved in the care of extremely preterm infants in 4 UK National Health Service perinatal centers between February 10, 2020, and April 30, 2021. Each participating center had a tertiary level neonatal unit and maternity center. Individuals volunteered participation through choosing to complete the study following a presentation by researchers at each center. A link to the online Q study was emailed to all potential participants by local principal investigators. Participants ranked 53 statements about the treatment of extremely preterm infants in an online quasi-normal distribution grid from strongly agree (6) to strongly disagree (−6).

**Main Outcomes and Measures:**

Distinguishing factors per professional group (representing different attitudes) identified through by-person factor analysis of Q sort-data were the primary outcome. Areas of shared agreement (consensus) between professional groups were also explored. Q sorts achieving a factor loading of greater than 0.46 (*P* < .01) on a given factor were included.

**Results:**

In total, 155 health care professionals volunteered participation (128 [82.6%] women; mean [SD] age, 41.6 [10.2] years, mean [SD] experience, 14.1 [9.6] years). Four distinguishing factors were identified between neonatal nurses, 3 for midwives, 5 for neonatologists, and 4 for obstetricians. Analysis of factors within and between professional groups highlighted significant variation in attitudes of professionals toward parental engagement in decision-making, the perceived importance of potential disability in decision-making, and the use of medical technology. Areas of consensus highlighted that most professionals disagreed with statements suggesting disability equates to reduced quality of life. The statement suggesting the parents’ decision was considered the most important when considering neonatal resuscitation was placed in the neutral (middistribution) position by all professionals.

**Conclusions and Relevance:**

The findings of this qualitative study suggest that parental counseling at extremely low gestations is a complex scenario further complicated by the differences in attitudes within and between professional disciplines toward treatment approaches. The development of multidisciplinary training encompassing all professional groups may facilitate a more consistent and individualized approach toward parental engagement in decision-making.

## Key Points

**Question:**

What are the attitudes of obstetric and neonatal health care professionals toward extremely preterm birth?

**Findings:**

In this qualitative study of 155 obstetric and neonatal health care professionals, significant variation and consensus were found in attitudes toward extremely preterm infants, both within and between professional groups involved in parental counseling. Differences were found in the prioritization of areas including parental involvement in decision-making, perceived infant outcomes, and the use of medical technology.

**Meaning:**

The findings of this study suggest that multidisciplinary training emphasizing reflexivity of health care professionals’ practice may be useful to facilitate a more consistent and individualized approach toward parental engagement and may reduce potential misalignment within and between professional groups.

## Teaser

This qualitative study examines the attitudes of obstetric and neonatal health care professionals regarding counseling parents and caring for extremely preterm infants.

## Introduction

### Objective

Despite improvements in perinatal and neonatal care, prematurity remains both the cause of the largest proportion of deaths in children younger than 5 years and a substantial contributor to adverse health outcomes in children and adults.^1,2,3,4^ In the UK, professional guidelines indicate management strategies for active and palliative care among infants born at 22 weeks of gestation or more, involving collaboration between senior clinical staff from obstetric, midwifery, and neonatal teams.^5^ Decision-making in these often rapidly developing scenarios, however, remains complex and emotionally challenging for health care professionals and parents.^6^ Studies have highlighted different interpretations of such guidance, and wide regional variation in infant outcomes of mortality and morbidity suggests variable approaches and biases of health care professionals during high-risk delivery.^7,8^ Such variation has implications for parental counseling and the informed engagement of parents in decision-making in situations in which prognosis is uncertain.^9^ Furthermore, variation in counseling practices between the different professional groups involved in preterm delivery has been identified, highlighting the potential for ambiguity and confusion among parents having multiple conversations with multiple professionals.^10^

Differing approaches by midwives, obstetricians, neonatologists, and nurses involved in perinatal counseling of women and families at extremely low gestational ages (<24 wk) have the potential to promote conflicting advice, particularly in situations in which opinions toward treatment decisions differ. Previous research has explored the attitudes of neonatal health care professionals toward the treatment of extremely preterm infants and highlights conflicting attitudes toward areas such as parental involvement, initiation of treatment, and the perceived impact of disability for the infants.^11,12,13^ Few studies have considered the attitudes of the midwifery and obstetric teams in these scenarios, despite their involvement in directing critically important maternal management in the delivery suite.^14^ These professionals are often the first to discuss with parents what is happening before consultation with the neonatal team, and thus the quality and consistency of both the approach toward and messages shared with parents across the whole perinatal team is crucial.^15,16^ Collaboration and communication between the neonatal and maternity teams ensure parents receive uniform, evidence-based information about their infants’ prospects for life, death, and disability. Supporting parental participation in treatment decisions enables safe and effective care and ensures and promotes optimal engagement with families.^17,18^

Understanding the breadth of attitudes toward the treatment of extremely preterm infants among different professionals would therefore have value and, to our knowledge, has not been undertaken previously. The aim of this study was to explore the attitudes of groups of health care professionals involved in the counseling of parents facing preterm birth and to compare attitudes within and between professional groups in order to understand how different factors associated with decision-making for extremely preterm infants are prioritized.

## Methods

The study aimed to assess the range of opinions within 4 professional groups. Neonatal nurses, midwives, neonatologists (subspecialist training level ≥4), and obstetricians (subspecialist training level ≥3) in 4 different UK National Health Service (NHS) perinatal centers around the UK were invited to participate in the study between February 10, 2020, and April 30, 2021. Participating centers had labor wards and tertiary level neonatal units, ensuring potential participants had the opportunity to meet study inclusion criteria of involvement in parental counseling and infant care management during extremely preterm birth. We aimed to recruit 10 participants per professional group at each participating center (40 participants per center) to facilitate factor analysis and enable between-group comparisons. The research team (K.G. and C.S.) presented the study at virtual grand rounds. Follow-up emails were sent via local principal investigators to all potential participants, containing patient information sheets and links to the online consent form and Q sort (ranking exercise). Demographic information was collected anonymously at the start of the Q sort (age, years’ experience, and sex) because sample sizes in Q method are small, limiting the ability to significantly link demographic details to results. Institutional research approval from the Health Research Authority was granted before the start of the study, along with approval from each perinatal centers’ research and development department. Voluntary informed consent from participants was gained through an online platform (SurveyMonkey) before completion of the survey. Participants did not receive financial compensation. This study followed the Standards for Reporting Qualitative Research (SRQR) reporting guideline.

### Q Methodology

The Q method facilitates the exploration of people’s opinions about a phenomenon by extracting statements from the literature and asking participants to rank them, either virtually or manually, from strongly agree to strongly disagree in a distribution grid, in a process known as Q sorting.^19,20^ Numerical data are gathered through the graduated labeling of statements from most agree (eg, +6) to most disagree (eg, −6). Factor analysis using a dedicated software package, PQMethod,^21^ is undertaken using individuals as grouping variables to determine where participants have placed statements in statistically significantly similar positions. The degree of association between individual participant Q sorts and factors determines which participants significantly identify with the viewpoint expressed by each factor. It is unlikely that all variance or viewpoints will be captured in the population and not all participants will load onto any single factor; rather, key viewpoints best representing the participant group are identified.^22^

Analysis identifies distinguishing statements within each factor; these statements reflect those that participants have placed in a significantly different position from that of other participants in other factors. Analysis also identifies consensus statements that all participants have placed in a similar position across all factors, which is useful in identifying shared viewpoints among all participants. Qualitative analysis of each factor is undertaken through interpretation of individual rankings and overall configuration of statements, developing a narrative reflecting each viewpoint. This process was undertaken independently by 2 of us (K.G. and C.S.) before discussion to finalize the results.

Q statements for this study were previously developed and studied with neonatal nurses and neonatologists independently.^11,12^ Statements were reviewed by the study team for relevance; all were deemed relevant and therefore kept to facilitate comparison with previous research. Fifty-three statements derived from thematic analysis represent statements in 6 categories: adverse outcomes, abortion limitations (within the UK, abortion is permitted until 24 weeks of gestation), involvement in decision-making, the use of advanced medical technology, treatment decisions, and fertility (eTable in the Supplement). Statements were entered into QSortware to enable online participation using a quasi-normal distribution grid, allowing participants to use their expert knowledge to prioritize at the extremes of the distribution.^22^

### Statistical Analysis

Data were extracted from QSortware and manually entered into and analyzed using PQMethod.^23^ Centroid factor analysis was undertaken to allow manual identification of association between Q sorts and factors. Q sorts that achieved factor loading of greater than 0.46 on a given factor (reaching significance at the *P* < .01 level) were included in that particular factor. Seven factors in each professional group were extracted before analysis to estimate the optimal number of factors best representing participants. Variance, participant numbers, and ability to interpret resulting factor arrays were considered.^22^ Data were analyzed per professional group to allow for within- and between-group comparisons of attitudes and to identify shared areas of agreement among all professionals.

## Results

In total, 155 professionals participated in the Q study (40 neonatal nurses, 40 neonatologists, 40 midwives, and 35 obstetricians). Of these, 128 (82.6%) were women and 27 (17.4%) were men; mean (SD) age was 41.6 (10.2) years and the mean (SD) time of experience was 14.1 (9.6) years. There were 10 participants per professional group per perinatal center, except for obstetricians—we were unable to recruit more than 5 from 1 facility. A range of factor solutions was identified across all professional groups (Table 1). These factor solutions resulted in the inclusion of the Q sorts of 70% of participating midwives, 63% of neonatal nurses, 60% of obstetricians, and 63% of neonatologists. The combined factor solutions of each professional group explained 53% of the variance for midwives, 60% among neonatal nurses, 55% for obstetricians, and 60% for neonatologists.

**Table 1.** Results of the Q Sort Analysis

Professional group and factor	Q sorts included, No. of participants	Variance explained by each factor, %
Midwives		
1	17	25
2	4	13
3	7	15
Neonatal nurses		
1	14	20
2	4	10
3	5	12
4	3	7
Neonatologists		
1	4	12
2	5	14
3	7	15
4	6	13
5	3	6
Obstetricians		
1	9	18
2	5	13
3	7	14
4	3	10

### Distinguishing Factors

Analysis identified different factor solutions for each professional group, each representing a different number of distinguishing statements (Table 1). Interpretation of the distinguishing statements identified a multitude of differing opinions both within and between professionals toward the treatment of extremely preterm infants. Exemplar statements reflecting all factors can be found in Table 2.

**Table 2.** Exemplar Statements and Their Respective Ranked Positions Representing Each Factor Retained for Analysis per Professional Group^a^

Group	Statement	Q sort value
**Midwives**		
F1 statements	6. The care of women in the neonatal unit should not be influenced by a history of previous abortions.	5
	14. Women should have the right to choose abortion up until 24 wk GA.	5
	39. Parents who do not want a disabled child should be able to make the decision to withhold or withdraw full intensive care treatment.	3
	35. Euthanasia protocols for extremely preterm infants should be introduced in the UK.	2
	51. Abortions should not be allowed from 22 wk GA as the fetus is changing into a baby.	−4
F2 statements	17. Infants born extremely preterm with life-limiting illness should still be given full intensive care treatment.	5
	3. HCPs should deliver the care that parents are asking for, even if parents are asking for treatment that HCPs think is futile.	4
	8. Infants born extremely preterm to families who have received IVF and are unlikely to conceive again should always be offered full intensive care treatment at all costs.	3
	44. Evidence of severe disability is a valid reason to withdraw treatment in an extremely preterm infant.	0
	1. Peaceful death is more important than full intensive care treatment.	−1
	10. The amount of technology used in the neonatal unit is a barrier that is detrimental to parent-infant bonding.	−3
	53. Technological developments mean that heroic measures of extraordinary means of support are overused.	−3
F3 statements	42. The most important factor when deciding on resuscitation is the potential of long-term suffering for the baby.	5
	25. The technology that enables extremely preterm infants to survive brings increased ethical dilemmas over whether it should be used to ensure this survival.	5
	24. The abortion limits should be reduced in accordance with the current limits of infant viability.	4
	5. The more disabilities that can be diagnosed prenatally, the more pressure there is on women to abort these pregnancies.	3
	3. HCPs should deliver the care that parents are asking for, even if parents are asking for treatment that HCPs think is futile.	−3
	35. Euthanasia protocols for extremely preterm infants should be introduced in the UK.	−5
**Neonatal nurses**		
F1 statements	42. The most important factor when deciding on resuscitation is the potential of long-term suffering for the baby.	6
	1. Peaceful death is more important than full intensive care treatment.	5
	11. If life-limiting disability is diagnosed prenatally, parents should be able to give birth to their child and enjoy the time they have without the option of full intensive care treatment.	5
	17. Infants born extremely preterm with life-limiting illness should still be given full intensive care treatment.	−2
	48. Parents should not be involved in treatment decisions for extremely preterm infants as they do not understand complex medical information.	−5
F2 statements	41. Better provision of welfare services in the community once children are older would make it easier to continue treatment for extremely preterm infants who display evidence of disability.	5
	8. Infants born extremely preterm to families who have received IVF and are unlikely to conceive again should always be offered full intensive care treatment at all costs.	5
	5. The more disabilities that can be diagnosed prenatally, the more pressure there is on women to abort these pregnancies.	4
	53. Technological developments mean that heroic measures of extraordinary means of support are overused.	3
	12. The most important factor when deciding on resuscitation is the potential burden on the parents.	−6
	26. Deciding whether to withhold or withdraw treatment is too stressful for parents and should be done by HCPs.	−5
F3 statements	24. The abortion limits should be reduced in accordance with the current limits of infant viability.	5
	38. The technology used on the neonatal unit allows more safety and control as the infants’ status is continually updated.	4
	50. Infants who are born alive following termination of pregnancy should be transferred to NICU for a trial of life.	4
	45. The current abortion limit of 24 wk GA is adequate, as infants <24 wk should not normally be resuscitated due to low survival rates and high risks of disability.	−5
	48. Parents should not be involved in treatment decisions for extremely preterm infants as they do not understand complex medical information.	−6
F4 statements	1. Peaceful death is more important than full intensive care treatment.	6
	12. The most important factor when deciding on resuscitation is the potential burden on the parents.	1
	26. Deciding whether to withhold or withdraw treatment is too stressful for parents and should be done by HCPs.	2
	28. Death is, and always will be, inevitable, for some infants.	0
	49. The choices that parents make about their extremely preterm infants are often prompted by the choices of the HCPs.	−4
	50. Infants who are born alive following termination of pregnancy should be transferred to NICU for a trial of life.	−6
**Neonatalogists**		
F1 statements	25. The technology that enables extremely preterm infants to survive brings increased ethical dilemmas over whether it should be used to ensure this survival.	5
	42. The most important factor when deciding on resuscitation is the potential of long-term suffering to the baby.	5
	44. Evidence of severe disability is a valid reason to withdraw treatment in an extremely preterm infant.	5
	10. The amount of technology used in the neonatal unit is a barrier that is detrimental to parent-infant bonding.	3
	43. Saving infants <24 wk of gestation is an inefficient use of NHS resources.	−3
	35. Euthanasia protocols for extremely preterm infants should be introduced in the UK.	−5
	4. Life should be maintained irrespective of outcome.	−6
F2 statements	11. If life-limiting disability is diagnosed prenatally, parents should be able to give birth to their child and enjoy the time they have without the option of full intensive care treatment.	6
	5. The more disabilities that can be diagnosed prenatally, the more pressure there is on women to abort these pregnancies.	5
	41. Better provision of welfare services in the community once children are older would make it easier to continue treatment for extremely preterm infants who display evidence of disability.	5
	34. Infant survival has become a secondary outcome, with determining how far technology can advance survival limits seemingly more important.	−3
	26. Deciding whether to withhold or withdraw treatment is too stressful for parents and should be done by HCPs.	−5
F3 statements	28. Death is, and always will be, inevitable, for some infants.	5
	44. Evidence of severe disability is a valid reason to withdraw treatment in an extremely preterm infant.	5
	48. Parents should not be involved in treatment decisions for extremely preterm infants as they do not understand complex medical information.	−2
	17. Infants born extremely preterm with life-limiting illness should still be given full intensive care treatment.	−3
	18. Full intensive care treatment should always be started as it can be withdrawn later if found to be futile.	−5
	3. HCPs should deliver the care that parents are asking for, even if parents are asking for treatment that HCPs think is futile.	−5
F4 statements	28. Death is, and always will be, inevitable, for some infants.	5
	14. Women should have the right to choose abortion up until 24 wk of gestation.	4
	44. Evidence of severe disability is a valid reason to withdraw treatment in an extremely preterm infant.	5
	49. The choices that parents make about their extremely preterm infants are often prompted by the choices of the HCPs.	3
	11. If life-limiting disability is diagnosed prenatally, parents should be able to give birth to their child and enjoy the time they have without the option of full intensive care treatment.	2
	45. The current abortion limit of 24 wk GA is adequate, as infants <24 wk of gestation should not usually be resuscitated due to low survival rates and high risks of disability.	−4
	50. Infants who are born alive following termination of pregnancy should be transferred to NICU for a trial of life.	−5
F5 statements	42. The most important factor when deciding on resuscitation is the potential of long-term suffering for the baby.	5
	45. The current abortion limit of 24 wk of gestation is adequate, as infants <24 wk of gestation should not usually be resuscitated due to low survival rates and high risks of disability.	4
	43. Saving infants at <24 wk of gestation is an inefficient use of NHS resources.	3
	19. Parents should be shown morbidity and mortality statistics following premature birth to help facilitate their decision-making.	3
	35. Euthanasia protocols for extremely preterm infants should be introduced in the UK.	−5
	3. HCPs should deliver the care that parents are asking for, even if parents are asking for treatment that HCPs think is futile.	−6
**Obstetricians**		
F1 statements	11. If life-limiting disability is diagnosed prenatally, parents should be able to give birth to their child and enjoy the time they have without the option of full intensive care treatment.	5
	14. Women should have the right to choose abortion up until 24 wk of gestation.	5
	45. The current abortion limit of 24 wk of gestation is adequate, as infants <24 wk should not usually be resuscitated due to low survival rates and high risks of disability.	3
	17. Infants born extremely preterm with life-limiting illness should still be given full intensive care treatment.	−2
	24. The abortion limits should be reduced in accordance with the current limits of infant viability.	−5
F2 statements	42. The most important factor when deciding on resuscitation is the potential of long-term suffering for the baby.	5
	24. The abortion limits should be reduced in accordance with the current limits of infant viability.	5
	49. The choices that parents make about their extremely preterm infants are often prompted by the choices of the HCPs.	4
	5. The more disabilities that can be diagnosed prenatally, the more pressure there is on women to abort these pregnancies.	3
	48. Parents should not be involved in treatment decisions for extremely preterm infants as they do not understand complex medical information.	−5
F3 statements	19. Parents should be shown morbidity and mortality statistics following premature birth to help facilitate their decision-making.	5
	41. Better provision of welfare services in the community once children are older would make it easier to continue treatment for extremely preterm infants who display evidence of disability.	4
	3. HCPs should deliver the care that parents are asking for, even if parents are asking for treatment that HCPs think is futile.	0
	36. NICU treatment accounts for a large proportion of NHS resources and, as such, admission of infants <24 wk of gestation should be restricted.	−5
	48. Parents should not be involved in treatment decisions for extremely preterm infants because they do not understand complex medical information.	−6
F4 statements	19. Parents should be shown morbidity and mortality statistics following premature birth to help facilitate their decision-making.	6
	44. Evidence of severe disability is a valid reason to withdraw treatment in an extremely preterm infant.	5
	35. Euthanasia protocols for extremely preterm infants should be introduced in the UK.	3
	23. There is a crossover between neonatal and abortion services due to limits of viability and legal limits of abortion.	3
	18. Full intensive care treatment should always be started because it can be withdrawn later if found to be futile.	0
	5. The more disabilities that can be diagnosed prenatally, the more pressure there is on women to abort these pregnancies.	−3
	30. The most important factor when deciding on resuscitation is the HCP’s opinion.	−4

Abbreviations: F, factor; GA, gestational age; HCP, health care professional; IVF, in vitro fertilization; NHS, National Health Service; NICU, neonatal intensive care unit.

^a^ Responses range from strongly agree (6) to strongly disagree (−6).

#### Midwives

All midwives were women (40 of 40 [100%]) with a mean (SD) age of 41.3 (12.3) years and 14 (11.2) years of experience. Three factors were identified. Seventeen of 40 midwives loaded onto factor 1. Statement positions reflected an attitude of respecting the choices of the woman (either pregnant or post partum) during the decision-making process, respecting the parents’ perspective of adverse outcomes while acknowledging the stress that medical technology can place on decision-making. Four of 40 midwives loaded onto factor 2. Statement positions reflected a more proactive application of medical technology to provide all opportunities for infant survival, with less emphasis on parental choice. Seven of 40 midwives loaded onto factor 3. Statements reflected an infant-centered approach to decision-making and the importance of considering adverse outcomes in this process.

#### Neonatal Nurses

Nearly all neonatal nurses (38 of 40 [95.0%]) were women, with a mean (SD) age of 39.2 (13.4) years and 14 (8.7) years of experience. Four factors were identified. Fourteen of 40 neonatal nurses loaded onto factor 1. Statement positions reflected the perceived importance of both short-term infant outcomes during decision-making and clinical opinion, while also considering parental choice in decision-making. Four of 40 neonatal nurses loaded onto factor 2. Statement positions reflected a stronger family involvement in decision-making, with less emphasis on outcomes. Five of 40 neonatal nurses loaded onto factor 3. Statement positions reflected a proactive approach to using medical technology to support infant survival, including infants born alive following termination of pregnancy, with less emphasis on outcomes or decision-making. Three of 40 neonatal nurses loaded onto factor 4. Statement positions reflected the prioritization of health care professionals’ opinion toward treatment, with statements prioritizing parental engagement through exploring outcome statistics, engagement with medical technology, or the decision-making process emphasized less.

#### Neonatologists

More than 60% of the neonatologists were women (25 of 40 [62.5%]) with a mean (SD) age of 43 (8.6) years and 12 (8.5) years of experience (39 neonatologists reported their years of experience). Five factors were identified. Four of 40 neonatologists loaded onto factor 1. Statements reflected decision-making, quality-of-life, and infant outcomes, and the ethical dilemmas that medical technology can bring to neonatal care. Five of 40 neonatologists loaded onto factor 2. Statement positions reflected a focus on adverse outcomes, although with a high prioritization of family involvement in decision-making and the provision of social support to facilitate this involvement. Seven of 40 neonatologists loaded onto factor 3. Statements reflected an acceptance of the current limitations of neonatal treatment, with less prioritization on parental involvement and higher prioritization on professionals’ treatment decisions. Six of 40 neonatologists loaded onto factor 4. Statement positions reflected an adherence to guidelines and decision-making while acknowledging the potential influence of health care professionals on parental decision-making. Three of 40 neonatologists loaded onto factor 5. Statement positions reflected professional treatment decisions when considering infant outcomes, with less prioritization on the decision-making process and parental involvement.

#### Obstetricians

More than 70% of obstetricians were women (25 of 35 [71.4%]), with a mean (SD) age of 43 (8.4) years and 17.5 (9.6) years of experience (33 obstetricians reported their experience). Four factors were identified. Nine of 35 obstetricians loaded onto factor 1. Statements reflected an agreement that abortion and neonatal services were separate entities and that decision-making in one should not influence the other, while recognizing the current limits of medical technology in neonatal care. Five of 35 obstetricians loaded onto factor 2. Statements reflected an agreement with the reduction in abortion limits to reflect current limits of viability with a stronger emphasis on parental engagement, the challenges parents face, and the consideration of infant outcomes. Seven of 35 obstetricians loaded onto factor 3. Statements reflected a considered approach to intervention, highlighting a clear opinion toward treatment decisions while acknowledging the importance of parental involvement. Three of 35 obstetricians loaded onto factor 4. Statements reflected a focus on parental engagement, although with an emphasis on the consideration of infant outcomes in decision-making.

### Areas of Consensus

Midwives had the highest number of statements indicating consensus (n = 18) followed by obstetricians (n = 15), neonatal nurses (n = 13), and neonatologists (n = 10) (Table 3). Areas of consensus were compared between professional groups to determine cross-disciplinary consensus. One of the 53 statements was identified as representing consensus across all professional groups, reflecting a neutral opinion (or nonprioritization) toward the level of importance of parents as ultimate decision makers during resuscitation. Six further areas of consensus were identified between at least 3 professional groups, reflecting attitudes toward disability, medical technology, and parental involvement in decision-making.

**Table 3.** Statements Identified as Areas of Consensus Across All Professional Groups, With Corresponding Ranked Positions for Each Factor^a^

Statement	Q sort value															
	Midwives			Neonatal nurses				Neonatologists					Obstetricians			
	F1	F2	F3	F1	F2	F3	F4	F1	F2	F3	F4	F5	F1	F2	F3	F4
7. It is wrong to knowingly bring a disabled child into this world.	−3	−5	−6	−3	−2	−4	−4	−5	−4	−4	−5	−4	NI	NI	NI	NI
13. Always initiating full intensive care treatment gives parents a chance to think that they have done everything they possibly could.	NI	NI	NI	1	0	0	1	1	2	0	0	1	1	−1	0	−1
16. Life satisfaction is not possible if you have a disability.	−5	−6	−5	−5	−5	−5	−5	−5	−5	−4	−4	−4	NI	NI	NI	NI
19. Parents should be shown morbidity and mortality statistics following premature birth to help facilitate their decision-making.	3	2	2	1	1	2	−1	2	1	2	1	3	NI	NI	NI	NI
21. The most important factor when deciding on resuscitation is the parents’ decision.	0	0	−1	−1	−1	−1	1	−1	−1	0	0	0	−1	2	1	0
27. Parents should be invited to learn about technology used on their extremely premature infant.	NI	NI	NI	2	1	1	−1	4	3	3	4	2	3	2	3	4
47. Caring has become technological, shifting the focus from caring for the infant to caring for the technology.	−1	0	−1	NI	NI	NI	NI	0	−1	0	−1	−1	−1	−1	−1	0

Abbreviations: F, factor; NI, not identified as an area of consensus by the respective professional group.

^a^ Responses range from strongly agree (6) to strongly disagree (−6).

## Discussion

This study identified areas of significant variation in attitudes toward extremely preterm infants within and between professional groups involved in parental counseling. Significant differences were found in the prioritization of statements toward areas including parental involvement in decision-making, the importance of perceived infant outcomes, and the use of medical technology. Differences were underpinned by shared areas of consensus indicating a high level of disagreement from midwives, neonatal nurses, and neonatologists with statements suggesting disability equates to reduced infant quality of life. Only 1 of the 53 statements was identified as representing consensus across all professional groups, highlighting participants’ neutrality (or nonprioritization) toward the suggestion that the parents’ decision was the most important when considering neonatal resuscitation.

The results of this study add to a growing body of literature highlighting differences in attitudes of obstetric and neonatal health care professionals involved in the complex scenario of extremely preterm birth.^24,25,26^ Similar to previous research, our findings suggest that some obstetricians may be less inclined toward intervention at the extremes of viability than some neonatologists, potentially as this area of care is less visible to obstetricians than neonatologists.^27,28^ As with previous research by some of us, we identified a more interventionalist approach among some neonatal nurses, potentially reflecting a gradual integration and acceptance of advanced medical technology that now forms part of the routine care of extremely preterm infants.^12^ For others, however, statements reflecting the ethical dilemmas that medical technology presented were more highly ranked, potentially reflecting situations whereby treatment is no longer limited to medical technology but includes the decision to apply this technology.^29^ Such differences in opinion could cause tension between teams and potentially affect parental counseling regarding initiation and continuation of treatment.^29^

All professional groups acknowledged the importance of parental engagement in decision-making; however, as with previous research, the degree of engagement varied.^26,30,31^ One area all participants agreed on was that, when considering resuscitation, the final decision should not reside with the parents alone. Perceptions of adverse outcomes appeared to underpin this variation, with statements reflecting the perceived effect of intensive care upon infants and evidence of disability in infants more often prioritized at the extremes of the distribution grid over statements highlighting parental involvement in decision-making. This reflects previous literature identifying that, particularly for neonatal nurses and neonatologists, perceived infant suffering and poor quality of life are distressing when caring for critically ill infants.^29,32^ Studies have consistently highlighted, however, the tendency for obstetricians, midwives, neonatologists, pediatricians, and neonatal nurses to underestimate survival and overestimate severe disability in extremely preterm infants.^33,34,35,36^ The unpredictable outcomes for these infants creates the potential for misalignment and miscommunication between professionals and between parents and professionals.^27^ Focusing decision-making on outcomes also potentially misses what is important to families.^37,38,39^ Studies conducted with younger people born extremely preterm and their parents explored outcomes important to them and showed that dichotomized outcome classifications are less important than how a child can participate in daily life activities, their social functioning, and mental health.^40,41^ Incorporating these elements into parental counseling at the margins of viability requires a cultural shift away from traditional outcome classifications.^42^

This study’s findings support multidisciplinary training among health care professionals to identify and discuss personal biases and develop teams that can communicate in a consistent and personalized manner with parents. Previous studies reported that intradisciplinary training in obstetric or neonatal care improves teamwork, and findings from the Morecambe Bay and Ockendon reports recommended multidisciplinary training to improve the overall safety of service provision.^17,43,44,45^ To our knowledge, however, there is minimal research to determine optimal approaches toward and outcomes from establishing multidisciplinary training and education for all professionals involved in extremely preterm birth. Effective training could foster a true sense of teamwork to allow health care professionals to identify how to provide optimal care to parents, ensuring parental views are heard and maintained at the forefront of interactions within such a large health care team. Such an approach may also result in fewer instances of miscommunication between professionals and parents from differences in estimations of mortality and morbidity and attitudes toward treatment.

Methods to facilitate this education require further research; currently, communication skills training for health care professionals involved in extremely preterm birth remains sporadic, voluntary, and in professional isolation.^46^ Recent training with intradisciplinary neonatal professionals around critical care decision-making used conversation analytic methods to facilitate reflection and discussion around real-life conversations.^47^ Such an approach using decision-making conversations between different professionals could potentially shed light onto this complex scenario and facilitate reflection and learning among all those involved.

### Limitations

This study has limitations. Results are potentially limited by sample size and are not intended to be generalizable to other perinatal centers; in the Q method the focus is less on large sample sizes and more toward understanding why and how people believe what they do.^48^ Although Q sorting is also self-directed, the narrative is limited by Q statement content; future research may identify further potential statements or outcomes relevant to decision-making. Because not all participants loaded significantly onto a factor, there is also potential for further influences that were not captured in this study. The results complement previous findings, however, and constitute what we believe to be the largest study of multidisciplinary attitudes toward preterm birth in England, warranting further educational research in this area.

## Conclusions

The differences in multidisciplinary attitudes toward extremely preterm birth noted in this qualitative study can lead to misalignment between health care professionals and cause confusion for parents trying to navigate this incredibly complex and emotional scenario. Multidisciplinary training involving all professionals involved is required that emphasizes health care professionals’ practice reflexivity, as well as communication style and strategy, to develop practices that facilitate consistent and personalized approaches toward parental engagement in decision-making.

## Notes

Supplement. **eTable.** Full List of Statements in the Q Sort
