# Supplementary material for: Attitudes About Extremely Preterm Birth Among Obstetric and Neonatal Health Care Professionals in England: A Qualitative Study
Source: JAMA Netw Open. 2022 Nov 14;5(11):e2241802. doi: 10.1001/jamanetworkopen.2022.41802 (PMC9664260; doi:10.1001/jamanetworkopen.2022.41802)
Supplement: Supplement. — eTable. Full List of Statements in the Q Sort [file jamanetwopen-e2241802-s001.pdf]

Supplementary Online Content

Gallagher K, Shaw C, Parisaei M, Marlow N, Aladangady N. Attitudes about extremely preterm birth among obstetric and neonatal health care professionals in England: a qualitative study. *JAMA Netw Open*. 2022;5(11):e2241802. doi:10.1001/jamanetworkopen.2022.41802

eTable. Full List of Statements in the Q Sort

This supplementary material has been provided by the authors to give readers additional information about their work.

© 2022 Gallagher K et al. *JAMA Network Open*

eTable. Full List of Statements in the Q Sort

**Statement**
1. Peaceful death is more important than full intensive care treatment
2. Advancing technology has made the process of withdrawing care more difficult
3. HCPs should deliver care that parents are asking for, even if parents are asking for treatment HCPs think is futile
4. Life should be maintained irrespective of outcome
5. The more disabilities that can be diagnosed prenatally, the more pressure there is on women to abort these pregnancies
6. The care of women in the neonatal unit should not be influenced by a history of previous abortions
7. It is wrong to knowingly bring a disabled child into this world
8 Infants born extremely prematurely to families who have received IVF and unlikely to conceive again should always be offered full intensive care treatment at all costs
9. Older parents are better equipped to deal with the outcomes of extreme prematurity
10. The amount of technology used in the neonatal unit is a barrier detrimental to parent-infant bonding
11. If life limiting disability is diagnosed prenatally, parents should be able to give birth to their child and enjoy the time they have without the option of full intensive care treatment
12. The most important factor when deciding on resuscitation is the potential burden on the parents
13. Always initiating full intensive care treatment gives parents a chance to think that they have done everything they possibly could
14. Women should have the right to choose abortion up until 24 weeks gestation
15. The amount of technology surrounding the infant alters the social concept of death to something that can be overcome
16. Life satisfaction is not possible if you have a disability
17. Infants born extremely preterm with life limiting illness should still be given full intensive care treatment
18. Full intensive care treatment should always be started as it can be withdrawn later if found to be futile
19. Parents should be shown morbidity & mortality statistics following preterm birth to help facilitate their decision making
20. Attempting to save babies <24w gestation is a large uncontrolled experiment
21. The most important factor when deciding on resuscitation is the parents decision
22. HCPs who work in abortion services from 20-24 weeks gestation are providing a service and should not be judged
23. There is a cross over between neonatal & abortion services due the limits of viability & legal limits of abortion (24 weeks gestation)
24. The abortion limits should be reduced in accordance with the current limits of infant viability
25. The technology which enables extremely preterm infants to survive brings increased ethical dilemmas over whether it should be used to ensure this survival
26. Deciding whether to withhold or withdraw treatment is too stressful for parents and should be done by HCPs

© 2022 Gallagher K et al. *JAMA Network Open*

27. Parents should be invited to learn about technology used on their extremely premature infant
28. Death is, and always will be, inevitable, for some infants
29. Parents are given a false sense of hope when they see all the equipment used on their extremely preterm infant
30. The most important factor when deciding on resuscitation is the HCPs opinion
31. Technology should be advanced to allow the most preterm infants to survive
32. Resuscitation <24w is for the parent's benefit only, not the baby's
33. Babies born <24w should always be resuscitated if the mother is too old to have any more children
34. Infant survival has become a secondary outcome, with determining how far technology can advance survival limits seemingly more important
35. Euthanasia protocols for extremely preterm infants should be introduced in the UK
36. NICU treatments account for a large proportion of NHS resources & as such admission of infants <24w should be restricted
37. It is better to have a disabled child, no matter how disabled, than no child at all
38. The technology used on the neonatal unit allows more safety & control as the infants status is continually updated
39. Parents who do not want a disabled child should be able to make the decision to withhold or withdraw full intensive care treatment
40. The philosophy underpinning nursing and medical care is the same in all health care settings, including neonatal & abortion services
41. Better provision of welfare services in the community once children are older would make it easier to continue treatment for extremely preterm infants who display evidence of disability
42. The most important factor when deciding on resuscitation is the potential of long-term suffering to the baby
43. Saving infants <24w is an inefficient use of NHS resources
44. Evidence of severe disability is a valid reason to withdraw treatment in an extremely preterm infant
45. The current abortion limit of 24w is adequate, as infants < 24w should not normally be resuscitated due low survival rates & high risks of disability
46. Women who try to conceive post menopause are not thinking about the best interests of the infant
47. Caring has become technological, shifting the focus from caring for the infant to caring for the technology
48. Parents should not be involved in treatment decisions for extremely preterm infants as they do not understand complex medical information
49. The choices that parents make about their extremely preterm infants are often prompted by the choices of the HCPs
50. 'Infants' who are born alive following termination of pregnancy should be transferred to NICU for a trial of life
51. Abortions should not be allowed from 22w as the fetus is changing into a baby
52. Abortion providers and NICUs are separate entities & the actions of one should have no influence upon the other
53. Technological developments mean that heroic measures of extraordinary means of support are overused

© 2022 Gallagher K et al. *JAMA Network Open*
